# Radiomic tumor phenotypes augment molecular profiling in predicting recurrence free survival after breast neoadjuvant chemotherapy

**DOI:** 10.1038/s43856-023-00273-1

**Published:** 2023-03-30

**Authors:** Rhea Chitalia, Marios Miliotis, Nariman Jahani, Spyros Tastsoglou, Elizabeth S. McDonald, Vivian Belenky, Eric A. Cohen, David Newitt, Laura J. van’t Veer, Laura Esserman, Nola Hylton, Angela DeMichele, Artemis Hatzigeorgiou, Despina Kontos

**Affiliations:** 1 Department of Bioengineering, University of Pennsylvania, Perelman School of Medicine 3400 Spruce Street, Philadelphia, PA 19104 USA; 2 Department of Radiology, Division of Hematology/Oncology, University of Pennsylvania, Perelman School of Medicine 3400 Spruce Street, Philadelphia, PA 19104 USA; 3 Department of Computer Science and Biomedical Informatics, University of Thessaly, Lamia, Greece; 4 DIANA-Lab, Hellenic Pasteur Institute, Athens, Greece; 5 Department of Radiology and Biomedical Imaging, University of California, San Francisco, USA; 6 Department of Surgery and Oncology, University of California, San Francisco, USA; 7 Department of Medicine, Division of Hematology/Oncology, University of Pennsylvania, Perelman School of Medicine 3400 Spruce Street, Philadelphia, PA 19104 USA

**Keywords:** Tumour biomarkers, Predictive markers

## Abstract

**Background:**

Early changes in breast intratumor heterogeneity during neoadjuvant chemotherapy may reflect the tumor’s ability to adapt and evade treatment. We investigated the combination of precision medicine predictors of genomic and MRI data towards improved prediction of recurrence free survival (RFS).

**Methods:**

A total of 100 women from the ACRIN 6657/I-SPY 1 trial were retrospectively analyzed. We estimated MammaPrint, PAM50 ROR-S, and p53 mutation scores from publicly available gene expression data and generated four, voxel-wise 3-D radiomic kinetic maps from DCE-MR images at both pre- and early-treatment time points. Within the primary lesion from each kinetic map, features of change in radiomic heterogeneity were summarized into 6 principal components.

**Results:**

We identify two imaging phenotypes of change in intratumor heterogeneity (*p* < 0.01) demonstrating significant Kaplan-Meier curve separation (*p* < 0.001). Adding phenotypes to established prognostic factors, functional tumor volume (FTV), MammaPrint, PAM50, and p53 scores in a Cox regression model improves the concordance statistic for predicting RFS from 0.73 to 0.79 (*p* = 0.002).

**Conclusions:**

These results demonstrate an important step in combining personalized molecular signatures and longitudinal imaging data towards improved prognosis.

## Plain language summary

Early changes in tumor properties during treatment may tell us whether or not a patient’s tumor is responding to treatment. Such changes may be seen on imaging. Here, changes in breast cancer properties are identified on imaging and are used in combination with gene markers to investigate whether response to treatment can be predicted using mathematical models. We demonstrate that tumor properties seen on imaging early on in treatment can help to predict patient outcomes. Our approach may allow clinicians to better inform patients about their prognosis and choose appropriate and effective therapies.

## Abstract (web summary)

Chitalia, Miliotis et al. evaluate radiomic predictors of outcome in patients with breast cancer treated with neoadjuvant chemotherapy. The authors identify radiomic phenotypes related to changes in tumor heterogeneity that improve progression-free survival prediction when added to clinicopathological and molecular factors.

## Introduction

Cancer is a dynamic and heterogeneous disease, with heterogeneity manifesting both across and within tumors^1,2^. Breast cancer heterogeneity specifically, is well-established, with intratumor heterogeneity arising due to genomic and transcriptomic variations leading to heterogeneous subpopulations driving prognosis and response to therapy^3,4^. As such, increased heterogeneity is thought to be associated with adverse clinical outcomes^5^.

Neoadjuvant chemotherapy (NACT) is an established course of treatment for locally advanced breast cancer (LABC) and can promote breast-conserving surgeries by reducing tumor size^6^. Additionally, women achieving pathologic complete response (pCR) after completing neoadjuvant chemotherapy may have improved survival outcomes^7,8^. Early prediction of response to neoadjuvant treatment can allow for personalized changes to treatment plans, including targeted therapies, and early discontinuation of inactive therapies^9,10^. Intratumor heterogeneity is thought to change in response to neoadjuvant chemotherapy leading to altered biomarker expressions^11^. Such changes may arise due to the acquired resistance by specific subclones during treatment^12^. Early, noninvasive characterization of such changes may indicate response versus resistance to treatment, enabling early treatment changes prior to treatment completion.

Personalized gene expression-based molecular assays, such as the 70-gene MammaPrint microarray assay (Agendia BV) and the 50-gene PAM50 risk of recurrence score assay (ROR-S), provide risk stratification for future recurrence^13,14^. p53 mutation status is an established predictor for more aggressive tumor biology and therefore a worse prognosis in terms of recurrence free survival (RFS)^15^. Such precision-medicine predictors may improve clinical decision-making by deviating from the “one size fits all” approach to treating breast cancer. However, as such assays, mutation statuses, and established histopathologic biomarkers are determined largely from selective tissue sampling acquired by biopsy, they may fall short in fully capturing heterogeneous disease burden.

Dynamic contrast-enhanced magnetic resonance imaging (DCE-MRI) can allow for longitudinal, non-invasive monitoring of heterogeneous tumors during the course of neoadjuvant chemotherapy. Previous studies have demonstrated the role of longitudinal patterns for tumor response during neoadjuvant chemotherapy and have examined their associations with treatment response and overall survival^16–19^. Hylton et al. demonstrated the prognostic and predictive value of measuring functional tumor volume (FTV) at various longitudinal time points during neoadjuvant chemotherapy^20^. Jahani et al. developed registration-based biomarkers for the early prediction of pCR and recurrence free survival (RFS) in tumors from baseline to early treatment time points^21^. While much progress has been made, these studies may be limited by not examining the associations between aggregate changes in intratumor heterogeneity that arise in response to therapy and the complementary information provided by genomics-based information^22^.

The purpose of this study was to identify imaging phenotypes of early changes in intratumor heterogeneity in DCE-MRI and evaluate their prognostic value in augmenting FTV measures and molecular profiling signatures scores for predicting RFS after breast NACT. We show that distinct heterogeneity imaging tumor profiles occur during neoadjuvant treatment for locally advanced breast cancer which can be utilized, in combination with personalized genomic biosignatures, to enhance current prognostic models and treatment management.

## Methods

### Discovery cohort

DCE-MR images of women enrolled in the ACRIN 6657/I-SPY1 trial, diagnosed with advanced invasive breast cancer from May 2002 through March 2006, were retrospectively analyzed^23,24^. Per the inclusion criteria of ACRIN 6657/I-SPY 1, women diagnosed with stage 2 or 3 breast cancer were selected for the study and underwent anthracycline-cyclophosphamide NACT. Longitudinal DCE-MRI was performed using a 1.5 T scanner at four time points: prior to the start of neoadjuvant therapy (T1), at least 2 weeks after the first cycle of chemotherapy (T2), between treatments (T3), and after the completion of chemotherapy, before surgery (T4). Data acquisition was as described in the ACRIN 6657/I-SPY 1 protocol^19^. The first and second post-contrast images were acquired 2.5 and 7.5 min after contrast injection.

Of the 222 trial participants with publicly available data^23,24^, we retained the 143 women for whom both complete clinical data and T1 and T2 DCE-MR imaging were available. For analyses involving gene expression, we used the subset of 100 women for whom gene expression information was available through the Gene Expression Omnibus^25,26^, under the accession number GSE22226^27^. Clinical and histopathologic data including age, hormone receptor (HR) status, human epidermal growth factor receptor 2 (HER2) status, and pCR status were available for each woman (Table 1). Functional tumor volume at T2 (FTV_2_), previously shown to have significant association with RFS^20^, was also calculated for each woman. RFS times were available, defined as time to recurrence (event), or time to death or last follow-up (censor).

**Table 1:** Selected patient characteristics for discovery cohort.

	No future event of recurrence (*n* = 72)	Future event of recurrence (*n* = 28)
Hormone Receptor positive	28 (53%)	17 (61%)
HER2 + positive	23 (32%)	11 (39%)
pCR	23 (32%)	4 (14%)
Age (min-max)	48.15 (33.18–64.33)	46.31 (28.76–65.39)

### Validation cohort

A validation cohort of 92 women was formed from the remaining 43 women from the original cohort (*n* = 143) for whom gene expression data was not publicly available, and a separate dataset of 49 women from the publicly available Breast MRI NACT Pilot study^28^. This study had similar inclusion criteria as the I-SPY 1 trial, and participants underwent a similar treatment and imaging protocol as the I-SPY 1 trial. Clinical information on age, HR status, and HER2 status and 3-year RFS information was available for each woman in the validation cohort (Supplementary Table 1).

### Research participants

All eligible patients selected for the I-SPY 1 TRIAL and Breast-MRI-NACT-Pilot study gave their written consent. In the I-SPY 1 TRIAL, the Health Insurance Portability and Accountability Act–compliant protocol and the written consent were approved by the American College of Radiology Institutional Review Board and local-site institutional review boards. More details regarding the trial’s study design and patients’ enrollment can be found here^29^. In the Breast-MRI-NACT-Pilot study, the research protocol was approved by an institutional review board (IRB). Details can be found here^28^. For this retrospective analysis, the requirement of informed consent was waived under institutional review board approval. Additional ethical approval for this retrospective study was not required as the data was publicly available and fully deidentified, hosted through the National Cancer Institute on the Cancer Imaging Archive^24^.

### Approximation of gene expression based molecular profiling signatures

Molecular profiling of the I-SPY 1 enrolled women with gene expression information was built as previously described^27^. Specifically, we re-created three gene signatures in order to classify tumors regarding their metastatic potential, risk of recurrence, and p53 oncogene mutation status: the 70-gene signature (MammaPrint)^30,31^, PAM50 risk of recurrence (ROR-S)^32,33^, and p53 mutation signature^34^ respectively. Briefly, MammaPrint classification was achieved by calculating the cosine similarity of the expression of the 70-gene signature for each sample against a “good prognosis” sample set^30^, using thresholds as defined in the original study^31^. ROR-S sample categorization was determined by computing the weighted sum of the correlation coefficients^33^ of each sample against the intrinsic subtype sample sets of the PAM50 gene signature study^32^. Lastly, p53 mutation status was estimated by calculating the proximity of the I-SPY 1 samples and the p53 mutation signature centroids (wildtype *vs*. mutant) as Spearman’s correlation values, as described in the p53 gene signature study^34^. The integrity of our classification was examined by comparing our results with the original results of the Esserman et al. study^27^. We confirmed that our recreated results corresponded to the original results by comparing the numbers of individuals attributed to each class in the overall cohort.

### Delta radiomic feature extraction

For each woman in the discovery cohort, the 3-D primary lesions at pre-treatment (T1) and early-treatment (T2) time points were selected by first identifying the functional tumor volume (FTV) within the publicly available bounding region, as previously reported^35^. The largest contiguous volume of voxels included in the FTV was selected as the location for the primary lesion; this volume was then further refined using manual segmentation to remove isolated voxels and include voxels within the primary tumor lesion volume which were not initially selected by the FTV threshold^35^. Final tumor segmentations for T1 and T2 were visually confirmed by a board-certified and fellowship trained breast imaging radiologist (ESM). Images were preprocessed by N3 bias-field normalization to correct for bias field signal^36^.

For each woman in the discovery cohort, at T1 and T2 time points, four voxel-wise kinetic image maps were calculated within the segmented tumor, the peak enhancement (PE) (Eq. 1), signal enhancement ratio (SER) (Eq. 2), wash-in slope (WIS) (Eq. 3), and wash-out slope (WOS) (Eq. 4) images, to quantify the enhancement patterns over the dynamic scans using the signal intensity for the pre-contrast, first post-contrast, and second post-contrast time points (*I*_0_, *I*_1_, and *I*_2_, respectively).

1 $$PE=\mathop{\max }\limits_{t={t}_{PE}}\frac{{I}_{t}-{I}_{0}}{{I}_{0}}$$

2 $$SER=\frac{{I}_{1}-{I}_{0}}{{I}_{2}-{I}_{0}}$$

3 $$WIS=\Bigg\{\begin{array}{cc}\frac{PE}{{t}_{PE}-{t}_{0}} & if\,{t}_{PE} \, \ne \, 0\\ 0 & otherwise\end{array}$$

4 $$WOS=\Bigg\{\begin{array}{cc}\frac{{I}_{2}-{I}_{1}}{{t}_{2}-{t}_{PE}} & if\,{t}_{2} \, \ne \, {t}_{PE}\\ 0 & otherwise\end{array}$$

All kinetic image maps and tumor segmentations were resampled by linear interpolation to a spatial resolution of 256 × 256 voxels, the lowest resolution of the data cohort, to ensure consistent resolution across all scans. A total of 104 radiomic features characterizing lesion intensity, texture patterns, and morphology were extracted from the entire tumor region, from each kinetic map at each treatment time point, resulting in a total of 416 features at each time point for each woman. All features were extracted using the publicly available Cancer Imaging Phenomics Toolkit (CaPTk; v.1.7.1; University of Pennsylvania; https://cbica.github.io/CaPTk/)^37^ (Supplementary Table 2). Features at each treatment time point (f_T1_ and f_T2_) were subsequently sign-adjusted such that increasing feature values corresponded to increasing lesion heterogeneity as per each feature definition. Subsequently, the change in each radiomic feature between the baseline and early treatment time points, or *delta feature* Δ*f*, was calculated as:

5 $$\varDelta f=\frac{{f}_{T2}-{f}_{T1}}{{f}_{T1}}$$

These delta features were subsequently z-score normalized and features with extreme skewness or low interquartile range (i.e., skewness > 5, IQR < 1) were excluded from further analysis. Features characterizing tumor texture or morphology in only 2-D image dimensions were also excluded to allow for whole-tumor, 3-D analysis. This resulted in a total of 42 delta features included in our final analysis. To reduce dimensionality and identify correlated delta features, features were clustered in an agglomerative hierarchical manner using Pearson’s correlation as the distance metric, with highly correlated features being grouped together. Consensus clustering was used to determine the optimal number of stable delta feature groups, with each feature group consisting of highly correlated delta features. Within each feature group, principal component analysis (PCA) was performed and principal components (PCs) totaling greater than 85% explained variance were retained to represent each feature group. As higher values for each delta radiomic feature prior to PCA indicated increasing heterogeneity from T1 to T2, higher values of a PC incorporating primarily positive contributions of features were interpreted as increasing heterogeneity, and one with negative contributions were interpreted as decreasing heterogeneity^38^. The PCs found, and their subsequent use in identifying imaging phenotypes of tumors, could serve to characterize tumors as having radiomic signatures indicating increasing or decreasing heterogeneity.

### Identifying imaging phenotypes of early change in tumor heterogeneity

To identify imaging phenotypes of early changes in tumor heterogeneity, tumors in the discovery cohort were classified via unsupervised hierarchical clustering, using the retained principal components to represent each tumor. The clusters identified through unsupervised clustering were interpreted as phenotypes of changes in heterogeneity seen in the study population. An overview schematic for how imaging phenotypes were generated can be found in Supplementary Fig. 1. An agglomerative hierarchical approach was used to cluster tumors, using Euclidean distance as the distance metric between the retained principal components for each tumor. Ward’s minimum variance method was used as the clustering metric^39^. To determine the optimal *k* number of clusters, consensus clustering^40^ was used to determine the number of stable phenotypes by repeatedly subsampling the data, performing unsupervised hierarchical clustering, and noting the proportion of subsamples in which, for every pair of tumors, they occupied the same cluster when they appeared in the same dataset. As such, a cumulative distribution function (CDF) was determined for each increase in *k*, and the stable number of clusters was determined to be the *k* at which the area under the CDF increased less than 10%. SigClust^41^ methods were used to determine the number of significant phenotypes by calculating the significance of the cluster index, a metric defined as the sum of within cluster sum of squares about the overall mean, tested against a null distribution at each cluster division. The significance of each phenotype split was tested at *p* < 0.05.

### Prognostic value of early change in heterogeneity phenotypes-statistical analysis

Distributions of clinical and histopathologic covariate values and molecular profiling scores were assessed for differences across radiomic phenotypes using Chi-square and Kruskal-Wallis tests for categorical and continuous covariates, respectively. Statistical corrections for multiplicity were made using the Bonferroni correction^42^.

RFS times across phenotypes were evaluated using Kaplan-Meier survival curves, in both the whole cohort and within strata of HR status, HER2 status, TN status, and greater than and less than median FTV_2_ values, with the log rank test used to determine statistical significance. RFS was also modeled via Cox proportional-hazards regression. Eight models were evaluated: univariable models for each molecular signature; the baseline model-using the covariates age, HR status, and HER2 status; baseline + FTV_2_; baseline + FTV_2_ + radiomic phenotype; and baseline + FTV_2_ + all molecular signatures, both with and without the addition of radiomic phenotype. All models were evaluated using 5-fold cross validation and averaged over 100 replicates.

The prognostic value of radiomic phenotypes was further evaluated by generating a risk score for each woman, defined as the prediction score of covariates weighted by the corresponding Cox-proportional hazard’s coefficients. Kaplan-Meier survival was analyzed split on the median risk calculated by the Cox model using baseline factors and FTV_2_.

Lastly, confusion matrices for the categories of RFS event/censor were generated to assess the predictive performance of radiomic phenotypes compared to MammaPrint scores, ROR-s, and p53 mutation status.

### Validation of early change in heterogeneity phenotypes

Tumor segmentations for cases in the validation cohort were generated similarly to those in the discovery cohort. Delta radiomic features were calculated using the same feature preprocessing methods used in the discovery cohort. The same delta features selected in the discovery cohort were also selected for the validation cohort. These resulting delta features were normalized using the mean and standard deviation values from the delta feature values in the discovery cohort, to standardize feature ranges.

Features were subsequently grouped together based on the cluster assignment of correlated features determined from the discovery cohort. Within each validation feature cluster, features were projected into the discovery cohort feature groups’-principal component space to determine component values. The same numbers of PCs summarizing each feature group retained in the discovery cohort were selected from the validation cohort to form the validation cohort principal-component vectors.

To determine phenotype assignment in the validation cohort, each tumor was assigned to the discovery cohort-identified phenotypes by minimizing the Euclidean distance between each validation cohort principal component vector and the discovery cohort phenotype centroid, defined as the average of principal component vector across all tumors in each phenotype.

### Reporting summary

Further information on research design is available in the Nature Portfolio Reporting Summary linked to this article.

## Results

### Discovery cohort

A total of 28 (28%) women included in the discovery cohort had future events of recurrence while 72 (72%) women did not have future events of recurrence (Table 1). Median RFS time was 3.9 years (range, 0.5–6.9 years)^35^. Neoadjuvant and radiation therapy information was available for women in the discovery cohort (Table 2).

**Table 2:** Selected treatment characteristics for discovery cohort.

Locally advanced cancers (*n* = 100)			
	No future event of recurrence (*n* = 72)	Future event of recurrence (*n* = 28)	*p*-value
Neoadjuvant Chemotherapy			> 0.99
Anthracycline-Cyclophosphamide (AC) only	1(1.4%)	0 (0%)	
AC + Tamoxifen	62 (86%)	24 (86%)	
AC + Tamoxifen + Herceptin	8 (11%)	3 (11%)	
AC + Tamoxifen + Other	1 (1.4%)	1 (4%)	
Herceptin	8 (11%)	3 (11%)	> 0.99
Radiation Therapy	58 (81%)	19 (68%)	0.21

### Validation cohort

Of the women included in the validation cohort, 27 (29%) women had future events of recurrence while 65 (71%) did not (Supplementary Table 1). Median RFS time was 4.13 years (range, 0.28–8.79 years).

### Gene expression signatures classification

Recreated classifications closely approximated the original results, considering minor differences regarding the sample cohorts (Supplementary Table 3–5). Following that, gene expression data were matched to the available imaging data for each patient. The recreated methods were then utilized to classify each tumor in the discovery cohort. Classifications are shown in Table 3. Further details regarding the recreated analysis are available in Supplementary Methods.

**Table 3:** Molecular profiles in the discovery cohort.

Gene signature	Distribution rates (*n* = 100)		
MammaPrint	7 (low risk)		93 (high risk)
p53 score	46 (wildtype)		54 (mutant)
PAM 50 ROR-S	31 (low risk)	31 (intermediate risk)	38 (high risk)

### Delta radiomic feature extraction

A total of four stable groups of correlated features was determined by consensus clustering. Selecting the PCs totaling greater than 85% explained variance from each group, a total of six principal components were identified to summarize change in heterogeneity for each primary lesion.

### Imaging phenotypes of early change in tumor heterogeneity

**Fig. 1:**
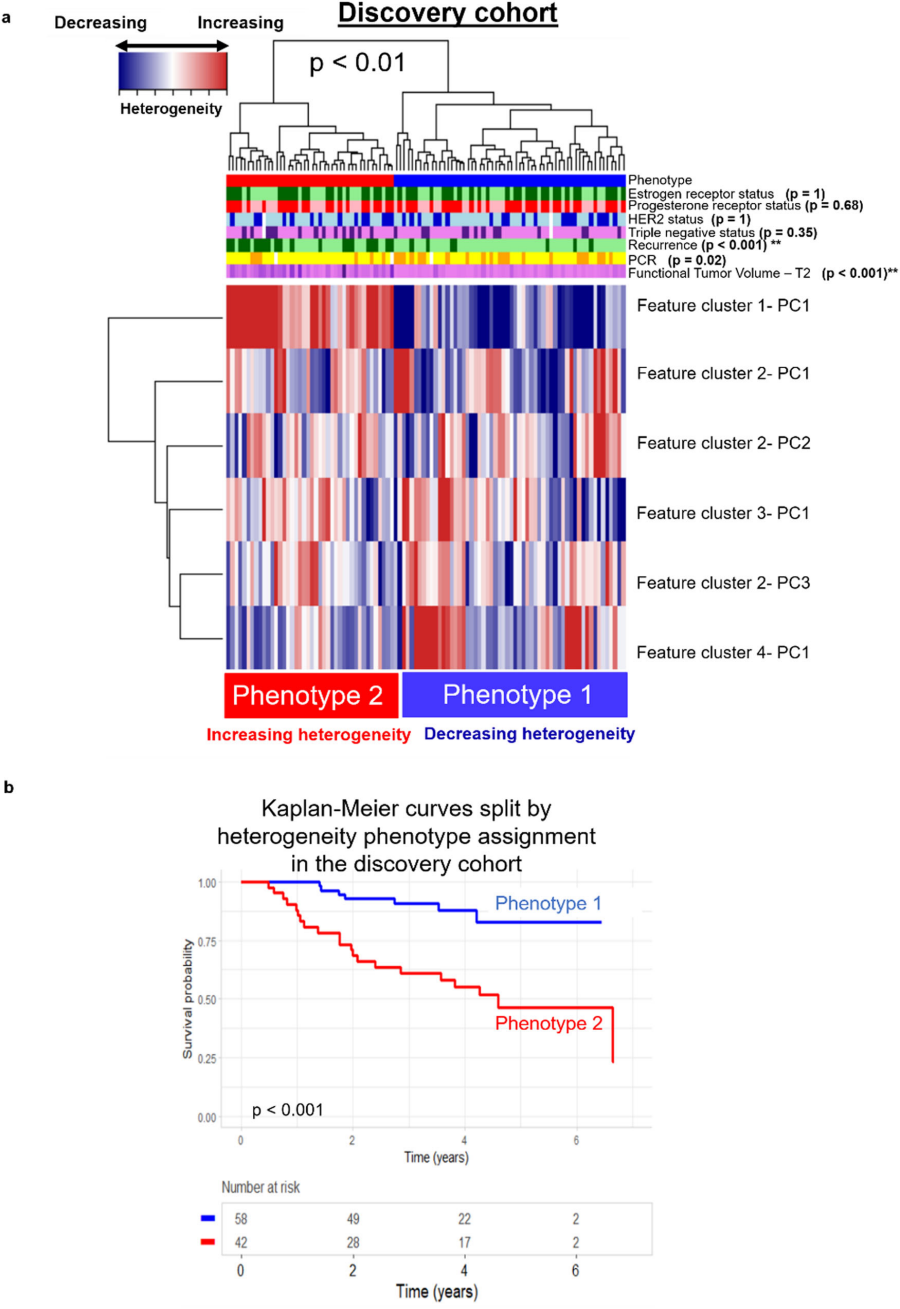
Unsupervised Hierarchical Clustering of Tumors in the Discovery Cohort. **a** Unsupervised hierarchical clustering of tumors in the discovery cohort (*n* = 100) identified two phenotypes of early changes in intratumor heterogeneity: decreasing heterogeneity from T1 to T2 (Phenotype 1, in blue) and increasing heterogeneity from T1 to T2 (Phenotype 2, in red). **b** Kaplan-Meier curves for recurrence free survival (RFS) of patient groups split by phenotype show significant separation, with tumors showing increase in intratumor heterogeneity after initiation of neoadjuvant therapy (Phenotype 2) having worse recurrence outcomes.

Two radiomic phenotypes of early change in intratumor heterogeneity were identified using unsupervised hierarchical clustering and shown to be statistically significant using the SigClust method (*p* < 0.01). Comparing the average of the six radiomic PC values observed for tumors in each phenotype allowed the two phenotypes to be interpreted as decreasing (Phenotype 1, *n* = 58) and increasing (Phenotype 2, *n* = 42) intratumor heterogeneity from T1 to T2 (Fig. 1). A Bonferroni statistical correction resulted in a *p*-value ≤ 0.007 to signify statistical significance in clinical covariate distribution across phenotypes. The number of future recurrences was significantly different across phenotypes (*p* < 0.001), with proportionally more recurrences in Phenotype 2 (increasing heterogeneity) than Phenotype 1 (decreasing heterogeneity), via the Chi-square test. Other clinical and histopathologic covariates, and molecular signatures, were not significantly different across phenotypes (Supplementary Fig. 2). Additionally, neoadjuvant treatment paradigms and targeted treatment paradigms were not significantly associated with radiomic phenotypes. Kaplan Meier RFS curves were also significantly different between phenotypes (*p* < 0.001).

**Fig. 2:**
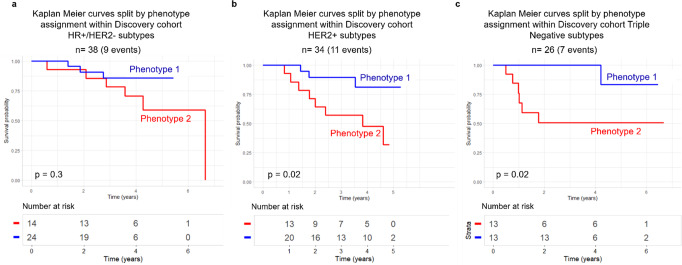
Survival Analysis for Molecular Subtypes of Breast Cancer. Kaplan Meier recurrence free survival (RFS) curves split by phenotype assignment for (**a**) HR + /HER2- (*n* = 38), (**b**) HER2 + (n = 34) and (**c**) Triple Negative (*n* = 26) molecular subtypes of breast cancer in the discovery cohort.

Within molecular subtypes of breast cancer, splitting women by radiomic phenotype assignment showed no significant difference in RFS for the HR + /HER2- subgroup (*p* = 0.3) and significant differences within the HER2 + and Triple Negative subtypes (both *p* = 0.02) (Fig. 2).

**Fig. 3:**
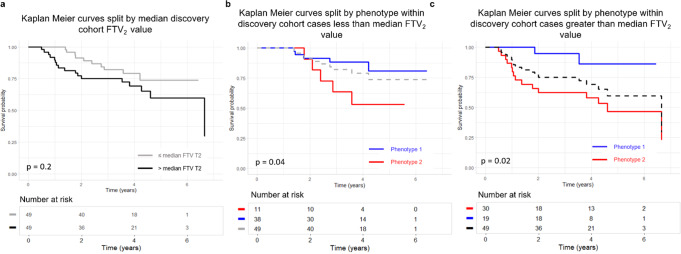
Survival Analysis for the Discovery Cohort Split by Median Functional Tumor Value. Kaplan Meier recurrence free survival (RFS) curves for the discovery cohort split by median functional tumor volume at T2 (FTV_2_) value (*n* = 100) (**a**) versus split by phenotype within strata of less than median FTV_2_ (*n* = 51) (**b**) and greater than median FTV_2_ (*n* = 49) (**c**). RFS split by above/below median FTV_2_ does not show *p* < 0.05 for separation. Within each stratum of FTV_2_, the split on phenotype is significant (**b** and **c**).

Kaplan Meier RFS curve separation by phenotype, within strata of FTV_2_ value (less than/greater than median FTV_2_) also demonstrated significant differences; curve separation on FTV_2_ itself was not significant (Fig. 3).

**Fig. 4:**
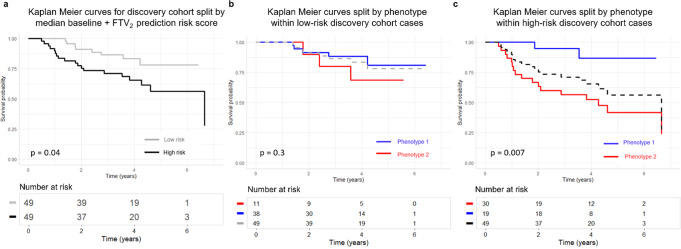
Survival Analysis versus Risk Score. Survival versus risk score for the discovery cohort calculated by a Cox model using baseline model covariates (age, HR status, and HER2 status) and functional tumor volume at T2 (FTV_2_). Split on above versus below median risk (*n* = 100) (**a**). **b** Split on phenotype within the low-risk stratum (*n* = 100). **c** Split on phenotype within the high-risk stratum (n = 100).

Kaplan-Meier curve separation of tumors split on the median risk score generated from a Cox proportional hazards model using baseline model covariates (age, HR status, and HER2 status) and FTV_2_ was significant (*p* = 0.04). Within the low-risk tumors, further separation on phenotype demonstrated no significant curve differences. For high-risk tumors, separation by phenotype was significant (*p* < 0.01) (Fig. 4).

Univariable Cox regression models based on each of MammaPrint, ROR-S, and p53 scores resulted in c-statistics of 0.63, 0.62, and 0.60, respectively. Kaplan Meier survival curves for MammaPrint, ROR-S, and p53 scores were not significant (Supplementary Fig. 3). A baseline model (model 1) based on age, HR status and HER2 status resulted in a cross validated, averaged over 100 replicates, c-statistic of 0.55. Adding FTV_2_ to the baseline model (model 2) improved the c-statistic to 0.67 and adding,molecular signatures to the baseline and FTV_2_ model (model 3) resulted in a c-statistic of 0.61. A model of baseline, FTV_2_, and radiomic phenotype assignment (model 4) resulted in a c-statistic of 0.73 and a combined model of baseline, FTV_2_, molecular profile scores, and radiomic phenotype assignment (model 5) demonstrated improved discriminatory capacity with a c-statistic of 0.79. The improvement in the final combined model was significant compared to the baseline, FTV_2_, and molecular signature score model, as determined by the log-likelihood test (*p* < 0.01) (Table 4).

**Table 4:** Univariable and multivariable Cox models of RFS within the discovery cohort.

Model	c-statistic	95% CI for c-statistic	Model *p*^a^	*p*-versus nested model
MammaPrint	0.63	0.57–0.69	0.2	
ROR-S	0.62	0.55–0.68	0.1	
p53 score	0.60	0.56–0.64	0.06	
Model 1: Baseline (age, HR status, HER2 status)	0.55	0.55–0.56	0.7	
Model 2: Baseline, FTV_2_	0.67	0.66–0.68	0.06	0.005^b^
Model 3: Baseline, FTV_2_, molecular signatures	0.61	0.59–0.62	< 0.05	0.13^c^
Model 4: Baseline, FTV_2_, phenotype	0.73	0.72–0.74	< 0.01	0.01^c^
Model 5: Baseline, FTV_2_, molecular signatures, phenotype	0.79	0.78–0.81	< 0.001	0.002^d^

^a^*p* versus null model of equal hazard for all patients.

^b^*p* versus Model 1, log-likelihood test.

^c^*p* versus Model 2, log-likelihood test.

^d^*p* versus Model 3, log-likelihood test.

**Fig. 5:**
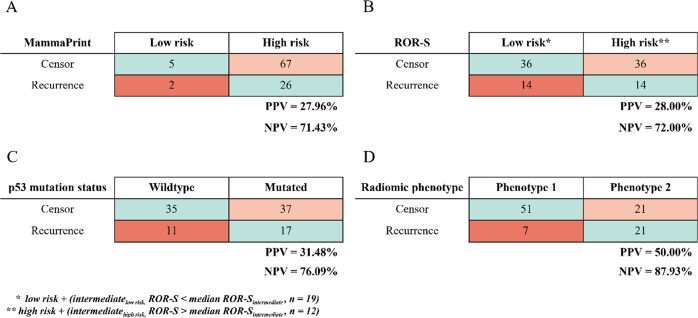
Confusion Matrices for Discovery Cohort. Confusion matrices for recurrence-free survival (RFS) prediction models within the discovery cohort using MammaPrint score (**A**), ROR-S (**B**), p53 mutation status (**C**), and radiomic phenotype (**D**).

Confusion matrices for associations between molecular profile scores and radiomic phenotypes and RFS event/censor were generated. Overall positive predictive values (PPV) and negative predictive values (NPV) for MammaPrint, ROR-S, p53 mutation status and radiomic phenotype assignment demonstrated that radiomic phenotype status had the highest PPV and NPV out of the four models (Fig. 5).

**Fig. 6:**
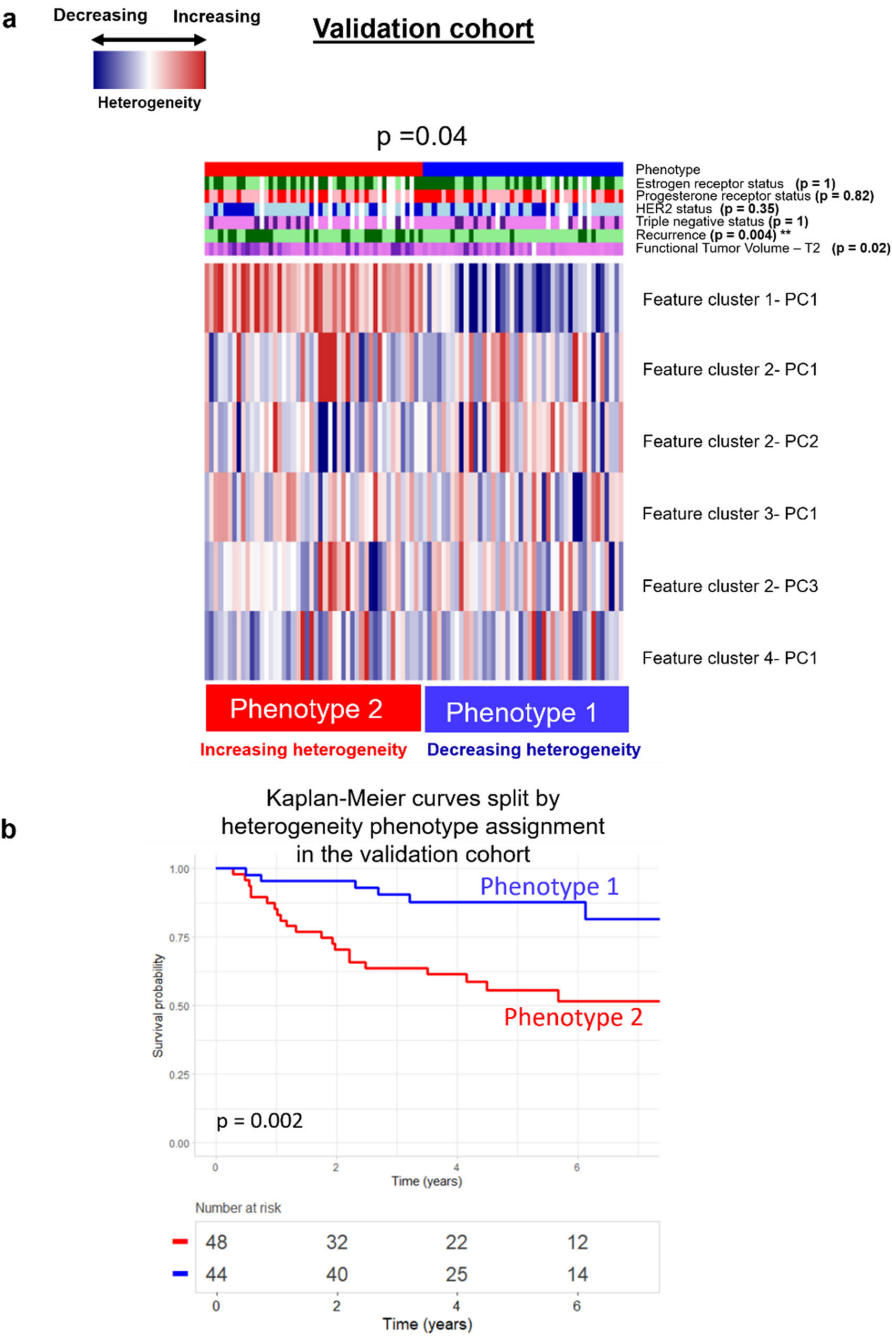
Replication of Phenotypes in the Validation Cohort. **a** Replication of radiomics phenotypes in the validation cohort found to be significant (*p* = 0.04). **b** Kaplan-Meier curves for recurrence free survival (RFS) split on radiomic phenotype show significant separation (*p* = 0.002).

The two radiomic phenotypes identified in the discovery set were replicated in the validation cohort and found to be statistically significant via the SigClust method (*p* = 0.04). Kaplan-Meier curves of tumors in the validation cohort split by phenotype also had a statistically significant difference (*p* < 0.01). A Bonferroni statistical correction calculated for the total number of comparisons being made resulted in a *p*-value < = 0.008 suggesting statistical significance in clinical covariate distribution across phenotypes. The proportional number of recurrences was significantly different across phenotypes (*p* = 0.004) using the Chi-square test, with Phenotype 2 (increasing heterogeneity) having proportionally more recurrence evens than Phenotype 1 (decreasing heterogeneity) (Fig. 6).

## Discussion

Two intrinsic radiomic phenotypes of early change in intratumor heterogeneity in response to neoadjuvant chemotherapy for locally advanced breast cancer were identified and validated. Interpretation of the two radiomic phenotypes as capturing an increase and decrease in intratumor heterogeneity from pre-treatment to early-treatment showed that tumors assigned to the phenotype with increasing intratumor heterogeneity had a greater number of future recurrences. This was further supported by significant separation in Kaplan Meier curves when stratifying women by phenotype assignment. Additionally, the stratification of women within FTV subgroups by phenotype demonstrates the added value of radiomic analysis in modeling prognosis (Fig. 3). Augmenting established clinical and histopathological prognostic factors with molecular signature scores and radiomic phenotypes resulted in better prediction of RFS. This suggests that leveraging the complementary information provided by genomic and radiomic data can allow for a more comprehensive assessment of tumors and personalized therapy selection.

There may be certain plausible explanations for our observations. By capturing changes in kinetic maps of the DCE-MRI data, the identified phenotypes could reflect changes in tumor composition and angiogenic properties in response to neoadjuvant chemotherapy. Increased heterogeneity may in turn reflect tumor plasticity, which can lead to acquired resistance. The imaging phenotype demonstrating increased heterogeneity from baseline to early-treatment exhibits an increased number of recurrence events, thus supporting the hypothesis that more heterogeneous tumors may result in more adverse clinical outcomes. In contrast, the radiomic phenotype demonstrating decreasing heterogeneity from pre-treatment to early-treatment included a higher number of tumors achieving pCR, which may suggest a relationship between decreased intratumor heterogeneity and an improved response to neoadjuvant chemotherapy (Fig. 1).

**Fig. 7:**
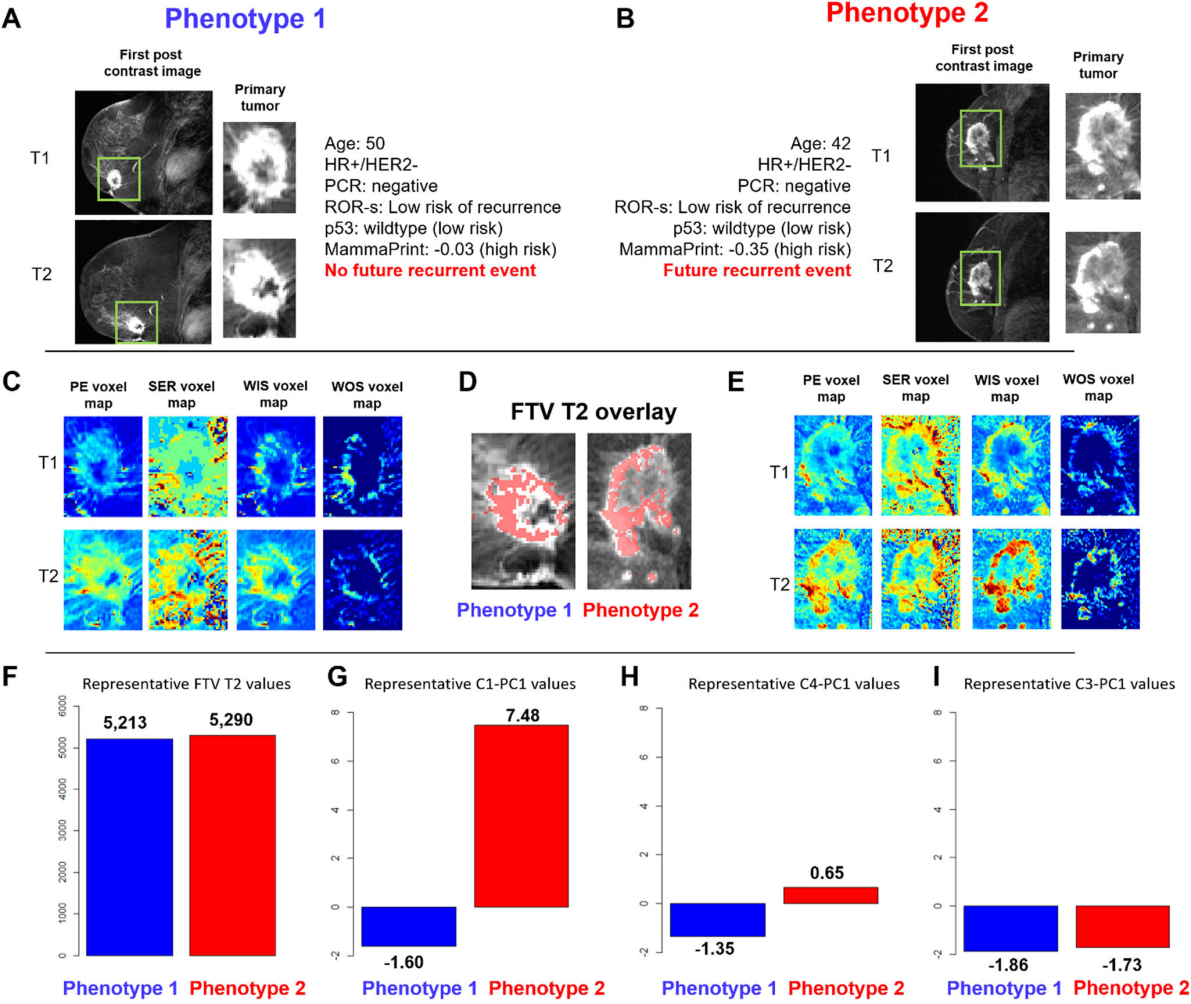
Representative Tumors from Each Phenotype. Representative tumors from Phenotype 1 (early decrease in intratumor heterogeneity) and. Phenotype 2 (early increase in intratumor heterogeneity) shown in DCE-MRI scans at T1 and T2 from the discovery cohort. **A** Representative 2D DCE-MRI slice and tumor region for T1 and T2 images from a woman, age 50, with an HR + /HER2-, ROR-S low risk, p53 wildtype (low risk), and MammaPrint score of −0.03 (high risk) tumor with no pCR and no future event of recurrence assigned to Phenotype 1. **B** Representative 2D DCE-MRI slice and tumor region for T1 and T2 images from a woman aged 42, with an HR + /HER2-, ROR-S low risk, p53 wildtype (low risk), and MammaPrint score of −0.35 (high risk) tumor with no pCR and a future event of recurrence assigned to Phenotype 2. **C** Representative 2D images of peak enhancement (PE), signal enhancement ratio (SER), wash-in slope (WIS), and wash-out slope (WOS) voxel-wise maps for T1 and T2 for the tumor in phenotype 1. **D** Functional tumor volume at T2 (FTV_2_) overlay for these representative tumors from phenotype 1 and 2. **E** Representative 2D images of PE, SER, WIS, and WOS voxel-wise maps for T1 and T2 for the tumor in phenotype 2. **F** FTV_2_ values for each representative tumor. Values for features (**G**) C1-PC1, (**H**) C4-PC1, and (**I**) C3-PC1, for each representative tumor. These representative cases provide an example where imaging characterizations of changes in each tumor’s heterogeneity provided a stratification related to future outcomes. In this example, established clinical covariates did not provide such stratification.

Interpreting the radiomic phenotypes of change in tumor heterogeneity through the lens of tumor biology may provide further insight into the biologic changes occurring within the tumor in response to neoadjuvant chemotherapy. As an example, two representative tumors from women with similar age, receptor status, FTV_2_ values, and genomic scores were assigned to separate imaging phenotypes based on their early change in heterogeneity. The tumor assigned to Phenotype 2, with an increase in intratumor heterogeneity after initiation of treatment, actually had a future event of recurrence while the tumor assigned to Phenotype 1, having a decrease in intratumor heterogeneity, did not have a future event of recurrence (Fig. 7). For these two representative cases, both women were of similar age with similar histopathologic status (HR + /HER2−). While ROR-S and p53 scores for both women characterized their tumors as “low risk of recurrence” and MammaPrint as “high risk of recurrence”, they were assigned to separate phenotypes based on their early change in their intratumor heterogeneity. In this particular example, the woman classified as “high risk” by MammaPrint score, went on to have no future recurrence event, while the woman classified as “low risk” based on ROR-S and p53 classifications did have a future event of recurrence. As none of the gene signature scores were significantly associated with phenotype assignment across the cohort (Supplementary Fig. 2), this suggests that the complementary information provided by radiomic and genomic analysis could allow for increased confidence in treatment planning and clinical decision-making. Furthermore, examining the principal component feature values for each woman suggests that quantitative imaging characterizations could reflect differences in these two tumors that may predict future outcomes. Of the six principal components used to cluster all tumors into the two phenotypes, C1-PC1, C4-PC1, and C3-PC1 distributions were found to be statistically significant tested against a *p*-value of 0.05 using Significance Analysis of Microarrays Test^43^. Examining the delta radiomics features comprising each feature cluster from which the principal components were generated could provide more insight into the specific quantitative differences in tumors in each phenotype. Specifically, as all radiomic features were extracted from the voxel-wise kinetic images, they provide a quantitative characterization of tumor angiogenesis and perfusion-related properties. C1-PC1 consists largely of features characterizing changes in tumor morphology across all kinetic images, including ellipse diameter and sphericity. In the representative images, the tumor assigned to Phenotype 2 has a greater value of this feature, suggesting that it had an increase in ellipse diameter and more irregular volume moving from T1 to T2. C4-PC1 consists of features characterizing changes in mean contrast intensity, specifically from the WOS image. As this image quantifies the rate of “wash-out” of contrast agent, the representative image in Phenotype 2 may have an increase in tumor wash-out from T1 to T2, suggesting an increase in leaky vasculature due to increased angiogenesis, a characteristic of more aggressive tumors^44^. Lastly, C3-PC1 consists of features summarizing morphologic flatness across all four kinetic images. Both representative tumors have similar values for this feature suggesting that both tumors decreased in morphologic flatness from T1 to T2 (Supplementary Table 6).

Significant separation of women by radiomic phenotype assignment by Kaplan-Meier curves for women with HER2 + and triple-negative breast cancers may further highlight the known sub-clonal diversity within these subtypes (Fig. 2)^45–47^. Our findings suggest that tumors within these subgroups that become more heterogeneous as an early response to neoadjuvant chemotherapy may be more aggressive, resulting in increased likelihood of recurrence.

Confusion matrices for RFS prediction using molecular signatures and radiomic phenotype assignment demonstrate a greater PPV and NPV when using radiomic phenotypes (Fig. 7). However, a limitation of using only radiomic phenotypes can be seen when comparing the predictive value of radiomic phenotypes alone against the MammaPrint assay. Seven women in Phenotype 1 went on to have recurrence despite decreasing heterogeneity on imaging whereas only 2 women, identified as a MammaPrint “low risk”, had a recurrence. Leveraging the complementary information from both personalized molecular signatures and incorporating longitudinal data about tumor heterogeneity resulted in the most accurate predictive model in our study.

Limitations to our study should be noted. First, our exploratory analysis included a relatively small sample size, as we restricted it to publicly available data from the ACRIN 6657/I-SPY 1 trial with both DCE-MRI and gene expression data available. In addition, the validation cohort utilized for this study did not include gene expression data which prevented us from validating the prognostic benefit of the molecular profiling scores. The publicly available microarray data used to generate the molecular profiling scores was also limited by older acquisition protocols and technology. Additionally, image analysis may have been limited by the older image acquisition protocol and technology used in the I-SPY 1 trial. However, the scan duration used for the dataset deriving from the I-SPY 1 trial was 4.5 min, which is similar to the current American College of Radiology (ACR) recommendation of < = 4 min^48^. Moreover, the datasets used in this study for discovery and validation are among the only publicly available datasets with true long-term follow-up available following NAC. Ultimately, given the encouraging results with these older MRI protocols, we can hypothesize that the performance of the proposed radiomic features may be better with newer MRI protocols. Future work will include expanding our analysis to larger cohort sizes with images acquired with newer, more clinically utilized MRI acquisition protocols, as well as exploring relationships between early changes in tumor heterogeneity via radiomic phenotyping and differentially expressed genes with related molecular pathways. Additionally, utilization of Next Generation Sequencing (NGS) techniques which, in contrast with microarrays, do not depend on specific probes for the quantification of the expression of pre-specified genes will allow for deeper and more rigorous analyses.

In conclusion, our exploratory results demonstrate that early changes in intratumor heterogeneity in response to neoadjuvant chemotherapy as captured by radiomic analysis of DCE-MRI may provide improved prediction of RFS for locally advanced breast cancer. Longitudinal non-invasive assessment of tumor phenotypes via imaging may allow for monitoring of heterogeneity and underlying tumor biology. Augmenting clinical, histopathologic, and molecular covariates with imaging phenotypes may allow for personalized risk stratification and early adaptation of treatment strategies.

## Supplementary information

Peer Review File

Supplementary Information

Reporting Summary

## Data Availability

The source data for all figures are available at 10.5281/zenodo.7327435^49^. All imaging data was available through The Cancer Imaging Archive^24^. Imaging data from the Discovery Cohort can be found listed as Multi-center breast DCE-MRI data and segmentations from patients in the I-SPY 1/ACRIN 6657 trials (ISPY1)^23,24^, and imaging data from the Validation Cohort can be found listed as Multi-center breast DCE-MRI data and segmentations from patients in the I-SPY 1/ACRIN 6657 trials (ISPY1) and as Single site breast DCE-MRI data and segmentations from patients undergoing neoadjuvant chemotherapy (Breast-MRI-NACT-Pilot)^28^. Gene expression information for the 100 women in the Discovery Cohort is available through the Gene Expression Ombinus^25,26^ under the accession number GSE22226^27^.

## Code availability

Analyses were conducted using R^50^. The code reproducing our findings is available at 10.5281/zenodo.7327435^49^.
