# Supplementary material for: Radiomic tumor phenotypes augment molecular profiling in predicting recurrence free survival after breast neoadjuvant chemotherapy
Source: Commun Med (Lond). 2023 Mar 30;3:46. doi: 10.1038/s43856-023-00273-1 (PMC10063641; doi:10.1038/s43856-023-00273-1)
Supplement: Supplementary file 2 — Supplementary Information [file 43856_2023_273_MOESM2_ESM.pdf]

Supplementary Information

Radiomic tumor phenotypes augment molecular profiling in predicting recurrence free survival after breast neoadjuvant chemotherapy

Rhea Chitalia *et al*.

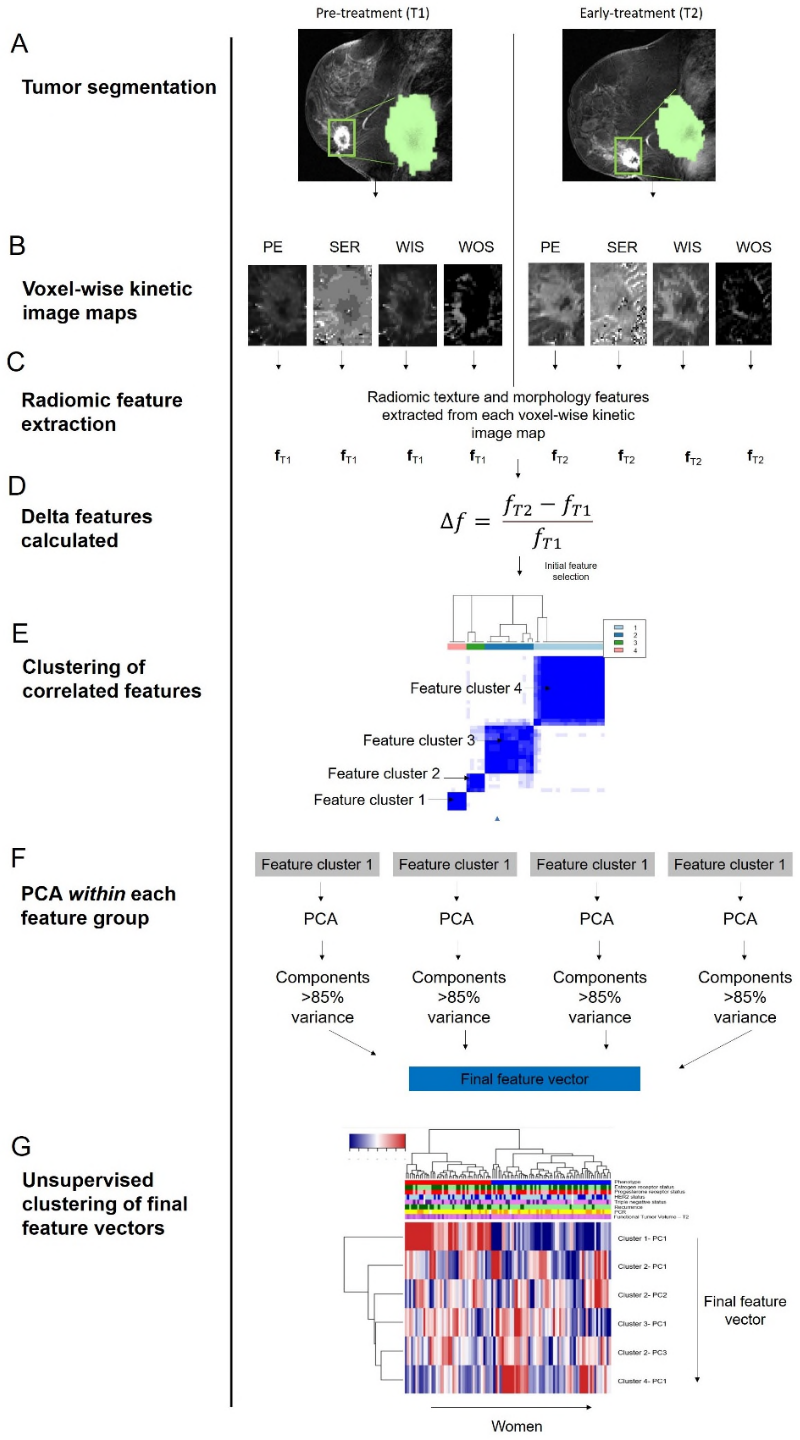

Supplementary Figure 1. Identification of change in heterogeneity phenotypes. (A) Primary lesion volume segmented from pre-treatment (T1) and early-treatment (T2) DCE-MRI images. (B) Four voxel wise kinetic image maps are created for peak enhancement (PE), signal enhancement ratio (SER), wash-in slope (WIS), and wash-out slope (WOS) images to quantify enhancement patters over each dynamic scan from T1 and T2 images. (C) Radiomic features are extracted from

each kinetic image map from both T1 and T2 images. (D) Delta radiomic features are calculated for each extracted feature. (E) Delta radiomic features are clustered based on correlation, and Consensus Clustering is used to determine the optimal number of stable feature clusters. (F) Within each feature cluster, PCA is performed, and the number of principal components is selected to account for >85% of explained variance. Selected principal components are concatenated across all feature clusters to form the final feature vector for each woman. (G) Unsupervised hierarchical clustering of the final feature vectors is performed to identify imaging phenotypes seen in the study population.

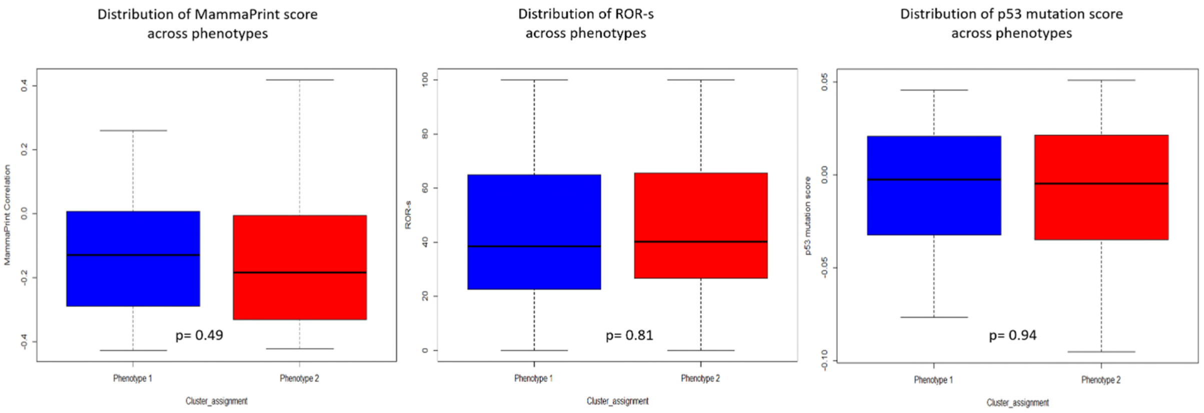

Supplementary Figure 2. Distribution of molecular profiling scores across phenotypes ($n_{Phenotype\ 1} = 42$, $n_{Phenotype\ 2} = 58$). Significant distributions across the phenotypes were not seen in the three molecular profiling scores. In each boxplot, the central line indicates the median, and the bottom and top edges of the box indicate the 25th and 75th percentiles, respectively. The whiskers extend to the most extreme data points not considered outliers. p53 mutation score corresponds to the absolute difference between the correlation of each sample against the reference p53 gene signature centroids (wildtype vs mutant).

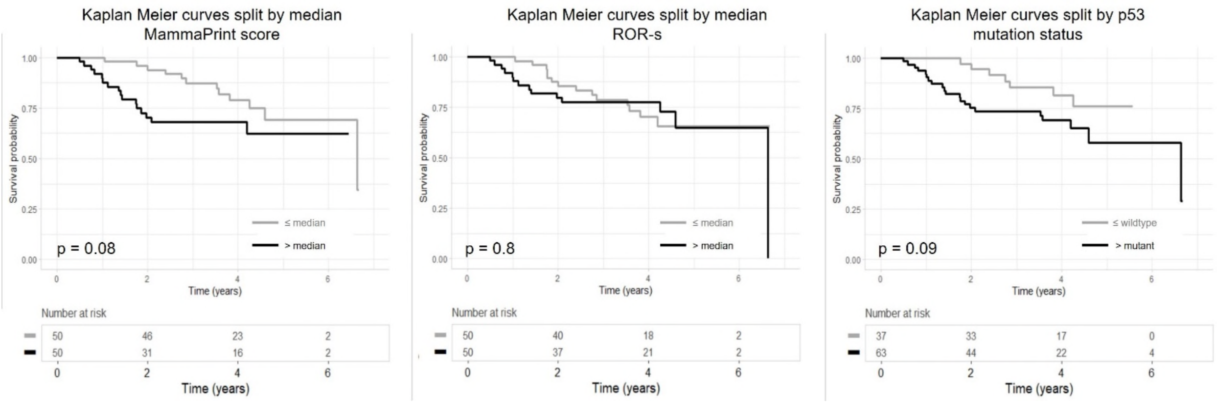

Supplementary Figure 3. Kaplan Meier survival curve for molecular profiling scores.

Supplementary Table 1: Selected patient characteristics for validation cohort.

	**No future event of recurrence (n=65)**	**Future event of recurrence (n=27)**
Hormone Receptor positive	37 (57%)	10 (37%)
HER2+ positive	19 (29%)	10 (37%)
pCR	16 (25%)	4 (15%)
Min. age	27.85	31.01
Max. age	71.47	63.8
Mean age	48.58	46.18

Supplementary Table 2: Radiomic feature families extracted from voxel-wise kinetic image maps.

**Feature Order**	**Feature Family**
First	Intensity
	Histogram
	Morphologic
	Volumetric
Second	Gray-level co-occurrence matrix
	Gray-level run-length matrix
	Gray-level size-zone matrix
	Neighborhood grey-tone matrix
Higher	Local binary pattern

Supplementary Table 3: Recreated MammaPrint classification results compared to the original Esserman et al. classification results.

	**Low risk**	**High risk**
Esserman's overall results	11	109
Recreated overall results	12	106
Esserman's PCR (positive) results	0/11	25/105
Recreated PCR (positive) results	0/12	28/106
Esserman's RCB (0 or I class) results	1/9	31/99
Recreated RCB (0 or I class) results	0/12	38/106

Supplementary Table 4: Recreated p53 mutation gene signature classification results compared to the original Esserman et al. classification results.

	**p53 wildtype**	**p53 mutant**
Esserman's overall results	59	61
Recreated overall results	57	61
Esserman's PCR (positive) results	5/58	20/58
Recreated PCR (positive) results	9/57	19/61
Esserman's RCB (0 or I class) results	10/53	22/55
Recreated RCB (0 or I class) results	14/57	24/61

Supplementary Table 5: Recreated PAM50 ROR-S gene signature classification results compared to the original Esserman et al. classification results.

	**Low risk**	**Intermediate risk**	**High risk**
Esserman's overall results	32	42	46
Recreated overall results	32	39	47
Esserman's PCR (positive) results	2/32	7/40	16/44
Recreated PCR (positive) results	3/32	11/39	14/47
Esserman's RCB (0 or I class) results	5/28	9/38	18/42
Recreated RCB (0 or I class) results	6/32	14/39	18/47

Supplementary Table 6 Radiomic features comprising significant feature cluster principle components.

**Cluster 1- PC1**	**Cluster 4- PC1**	**Cluster 3- PC1**
PE Intensity Quartile Coefficient of Variation	WOS Histogram Ninetieth Percentile	PE Morphologic Flatness
PE Morphologic Ellipse Diameter Axis 0	WOS Histogram Root Mean Square	SER Intensity Interquartile Range
PE Morphologic Ellipse Diameter Axis 1	WOS GLCM Entropy	SER Morphologic Flatness
PE Morphologic Ellipse Diameter Axis 2	WOS GLRM Short Run High Grey Level Emphasis	WIS Morphologic Flatness
PE Morphologic Equivalent Spherical Radius	WOS GLSZM Grey Level Mean	WOS Morphologic Flatness
SER Morphologic Ellipse Diameter Axis 0		
SER Morphologic Ellipse Diameter Axis 1		
SER Morphologic Ellipse Diameter Axis 2		
SER Morphologic Equivalent Spherical Radius		
WIS Intensity Quartile Coefficient of Variation		
WIS Morphologic Ellipse Diameter Axis 0		
WIS Morphologic Ellipse Diameter Axis 1		
WIS Morphologic Ellipse Diameter Axis 2		
WIS Morphologic Equivalent Spherical Radius		
WOS Morphologic Ellipse Diameter Axis 0		
WOS Morphologic Ellipse Diameter Axis 1		

WOS Morphologic Ellipse Diameter Axis 2	
WOS Morphologic Equivalent Spherical Radius	
WOS GLSZM Zone Size Nonuniformity	

Supplementary Methods

Gene expression data were obtained from Gene Expression Omnibus (GEO)[1,2] using the publicly available samples from the Esserman et al. study (accession GSE22226)[3] that match the ACRIN 6657/I-SPY 1 MRI data of the discovery cohort TCIA dataset. Samples have been analyzed using two microarray platforms and can be found in GEO under accessions GPL1708 (n = 130) and GPL4133 (n = 20). Initially, gene signature sets were validated by recreating the Esserman et. al study. Afterwards, the final gene signatures set were used solely and in combination with the MRI information of patients to assess their classifying value.

Recreation of Esserman et al. study

Samples were filtered as in Esserman et al.[3] down to n = 120 (initially 121, but one entry was removed due to incomplete data). Briefly, only patients with both microarray expression data and HR/HER2 status, RCB and negative *trastuzumab* treatment status were kept in the final set. *Trastuzumab* status was taken directly from the GEO phenotype information, while RCB class and HR/HER2 status were extracted using the Clinical and Outcome Data found at https://wiki.cancerimagingarchive.net/display/Public/ISPY1. Minimal differences in clinical data may be explained due to different updates in the various data sources, as the ACRIN 6657/I-SPY 1 clinical trial was an extended, long-term prospective study. Microarray intensity values provided in GSE22226 are expressed as log_2 Lowess-normalized mean ratio values.

Molecular profiling was built using three of the four gene signatures that are mentioned in Esserman et al. study i.e. 70-gene signature (MammaPrint)[4,5], p53 mutation signature[6] and PAM50 risk of recurrence (ROR-S)[7,8]. The wound-healing response gene signature[9] recreation proved to be unsuccessful due to unavailability of the original supplementary data and microarray probes that comprise the signature gene set. Annotation of the probes was extracted automatically from the latest GEO platform annotation found in the NCBI GEO repository.

MammaPrint

The original experiment consisted of 98 primary breast cancer human samples, of which 78 were metastasis-free. 44/78 were recurrence-free for more than 5 years, constituting the "good prognosis group". In short, the authors performed hierarchical clustering using significantly regulated genes (two-fold change, $p < 0.01$), Pearson correlation coefficient calculation between the prognostic category and the log expression ratio across all samples and Monte Carlo randomization of the association between the expression ratio and prognosis category to discern the best candidate genes (n = 231) to predict recurrence-free survival (RFS). After leave-one-out cross validation for different subsets of genes, the authors ended up with the 70-gene signature.

Classification is achieved by calculating the cosine similarity between the MammaPrint gene signature expression values of the sample to be classified and the average of the MammaPrint gene signature expression values of the "good prognosis group" in the original study. A value greater

than -0.4 (threshold suggested by the MammaPrint authors) is considered to classify the sample as "high risk" with respect to future recurrence[5].

The MammaPrint 70-gene signature consists of 70 microarray probes. Original analysis was performed using 25K human oligonucleotide two-color microarrays developed by Rosetta. The 70 probes correspond to 56 genes and 14 Expressed Sequence Tags (ESTs). Expression values provided in supplementary data are calculated as log_{10} mean ratio with median background intensity subtraction. Specifically:

$$expression\ value = \ log_{10} \frac{Channel_2 mean\ intensity - median(background\ intensity)}{Channel_1 mean\ intensity - normalized\ median(background\ intensity)}$$

In our study, the cosine similarities were calculated using the genefu R package[10]. Probe matching between Esserman et al. microarray annotation and MammaPrint was done using Entrez IDs, resulting into a shrinkage of the probes number (52/70). A possible explanation for this is due to updated annotation information related to the genes overlapping those probes. Furthermore, in order to make the two platform intensities comparable, the Esserman et al. expression values were recalculated to match the MammaPrint values. $-\infty$ and NA values (introduced due to background subtraction) were converted to 0 prior to cosine similarity estimation. MammaPrint classification results between the two studies are presented in **Supplementary Table 3**.

p53 mutation signature

The p53 mutation gene signature consists of 52 microarray probes derived from the unsupervised clustering of datasets with known p53 mutation status, which is used to classify samples' status as p53 wildtype or p53 mutant. Tumor suppressor p53 mutations are found more frequently in aggressive breast cancers. In the original study, a SAM[11] derived gene list along with a false discovery rate of less than 5% was given as input to an average linkage hierarchical cluster analysis. The analysis was conducted using Pearson correlation in the Cluster program. This gene list was then refined by comparing the p53-associated gene lists between tumor samples and cell lines, leading to a robust list of 52 genes that were common to both data sets (*in vitro* and *in vivo*).

According to Troester et al.[6], classification was performed by calculating the Spearman's correlation metric between the samples under examination and the training set centroids, similar to the MammaPrint method. Centroids are represented as vectors of the mean average of each gene expression in the p53 signature, one per p53 status group (wildtype *vs.* mutant). Greater correlation to one of the centroids classifies the sample as such. As such, the p53 mutation score corresponds to the absolute difference between the correlation of each sample against the reference p53 gene signature centroids (wildtype vs mutant)[6].

The training dataset used to create the centroids consists of 66 microarray samples from Sorlie et al. [12] (2001), which are deposited in GEO under accession GSE3193. In the experiment, 4 different microarray platforms were used i.e. GPL180, GPL2776, GPL2777, GPL2778. Microarray platform/source systematic biases between them were originally corrected using the Distance

Weighted Discrimination (DWD) algorithm. Due to lack of an R package that implements DWD, in our study we used the ComBat function found in the SVA R package[13], which utilizes an Empirical Bayes approach. Three groups were designated for batch removal (A: Sorlie et al. (2001) data, B: Esserman's GPL1708 data, C: Esserman's GPL4133 data). Non-finite values were ignored.

Due to absence of correspondence between outdated GenBank gene IDs in Troester et al.[6] and current Entrez gene IDs, manual curation was performed. This procedure resulted in a common set of 51/52 probes between all datasets, which comprised the final p53 gene signature used for the recreation analysis. The same rationale as in the MammaPrint section applies here: log_{10} and log_2 mean ratio and median background intensity subtraction were used in the different Sorlie et al. (2001) microarray datasets, thus every raw intensity was transformed into log_2 mean ratio values, including the microarray intensity values from Esserman et al.. p53 gene signatures results between our study and Esserman's are compared in **Supplementary Table 4**.

PAM50 ROR-S

Risk of recurrence score is used to classify patients into three categories (low, medium and high risk of relapse) according to an estimated risk value, using predefined thresholds. The original study progressively identified a 50-gene set through hierarchical clustering and the single sample predictor algorithm (SSP), which was used to cluster 189 breast cancer and 29 normal samples into 5 intrinsic subtypes (Luminal A, Luminal B, HER2-enriched, Basal-like and Normal-like) by employing the Prediction Analysis of Microarray (PAM) centroid-based clustering algorithm. Distance to each subtype was calculated using Spearman's rank correlation, representing the proximity of each sample to each category. Then, the authors performed univariate and multivariate analyses to determine the significance of those subtypes and trained a multivariable Cox model using the Ridge regression fit to the node-negative, untreated subset of the van de Vijver cohort[14]. In order to classify a sample into one of the risk categories, one needs to calculate the resulting weighted sum of the intrinsic subtype Spearman's rank coefficients using the following equation:

$$ROR - S = 0.05 * basal + 0.12 * HER2 + (-0.34 * LumA) + 0.23 * LumB$$

We utilized the genefu R package (rorS function) to classify our samples. Probe matching between the two gene sets was successfully done using Entrez IDs, which resulted in a complete 50/50 annotation. As in MammaPrint, the two platforms' intensities were incomparable. Thus, the Esserman et al. intensities were recalculated with no background subtraction and no Lowess normalization and expressed using log_2 mean ratios. Predefined ROR-S thresholds were used i.e. low $\leq$ 29, 29 $<$ moderate $<$ 53, high $\geq$ 53. ROR-S classification results for the two studies can be found in **Supplementary Table 5**.

Supplementary References

1 Edgar, R., Domrachev, M. & Lash, A. E. Gene Expression Omnibus: NCBI gene expression and hybridization array data repository. *Nucleic Acids Res* **30**, 207-210, doi:10.1093/nar/30.1.207 (2002).

2 Barrett, T. *et al.* NCBI GEO: archive for functional genomics data sets--update. *Nucleic Acids Res* **41**, D991-995, doi:10.1093/nar/gks1193 (2013).

3 Esserman, L. J. *et al.* Chemotherapy response and recurrence-free survival in neoadjuvant breast cancer depends on biomarker profiles: results from the I-SPY 1 TRIAL (CALGB 150007/150012; ACRIN 6657). *Breast Cancer Res Treat* **132**, 1049-1062, doi:10.1007/s10549-011-1895-2 (2012).

4 van 't Veer, L. J. *et al.* Gene expression profiling predicts clinical outcome of breast cancer. *Nature* **415**, 530-536, doi:10.1038/415530a (2002).

5 Glas, A. M. *et al.* Converting a breast cancer microarray signature into a high-throughput diagnostic test. *BMC Genomics* **7**, 278, doi:10.1186/1471-2164-7-278 (2006).

6 Troester, M. A. *et al.* Gene expression patterns associated with p53 status in breast cancer. *BMC Cancer* **6**, 276, doi:10.1186/1471-2407-6-276 (2006).

7 Hu, Z. *et al.* The molecular portraits of breast tumors are conserved across microarray platforms. *BMC Genomics* **7**, 96, doi:10.1186/1471-2164-7-96 (2006).

8 Parker, J. S. *et al.* Supervised risk predictor of breast cancer based on intrinsic subtypes. *J Clin Oncol* **27**, 1160-1167, doi:10.1200/JCO.2008.18.1370 (2009).

9 Chang, H. Y. *et al.* Robustness, scalability, and integration of a wound-response gene expression signature in predicting breast cancer survival. *Proc Natl Acad Sci U S A* **102**, 3738-3743, doi:10.1073/pnas.0409462102 (2005).

10 Gendoo, D. M. *et al.* Genefu: an R/Bioconductor package for computation of gene expression-based signatures in breast cancer. *Bioinformatics* **32**, 1097-1099, doi:10.1093/bioinformatics/btv693 (2016).

11 Tusher, V. G., Tibshirani, R. & Chu, G. Significance analysis of microarrays applied to the ionizing radiation response. *Proc Natl Acad Sci U S A* **98**, 5116-5121, doi:10.1073/pnas.091062498 (2001).

12 Sørlie, T. *et al.* Gene expression patterns of breast carcinomas distinguish tumor subclasses with clinical implications. *Proc Natl Acad Sci U S A* **98**, 10869-10874, doi:10.1073/pnas.191367098 (2001).

13 Leek, J. T., Johnson, W. E., Parker, H. S., Jaffe, A. E. & Storey, J. D. The sva package for removing batch effects and other unwanted variation in high-throughput experiments. *Bioinformatics* **28**, 882-883, doi:10.1093/bioinformatics/bts034 (2012).

14 van de Vijver, M. J. *et al.* A gene-expression signature as a predictor of survival in breast cancer. *N Engl J Med* **347**, 1999-2009, doi:10.1056/NEJMoa021967 (2002).
