# Supplementary material for: Radiomic tumor phenotypes augment molecular profiling in predicting recurrence free survival after breast neoadjuvant chemotherapy
Source: Commun Med (Lond). 2023 Mar 30;3:46. doi: 10.1038/s43856-023-00273-1 (PMC10063641; doi:10.1038/s43856-023-00273-1)
Supplement: Supplementary file 3 — Reporting Summary [file 43856_2023_273_MOESM3_ESM.pdf]

nature portfolio

Corresponding author(s): Rhea Chitalia

Last updated by author(s): Jan 31, 2023

Reporting Summary

Nature Portfolio wishes to improve the reproducibility of the work that we publish. This form provides structure for consistency and transparency in reporting. For further information on Nature Portfolio policies, see our Editorial Policies and the Editorial Policy Checklist.

Statistics

For all statistical analyses, confirm that the following items are present in the figure legend, table legend, main text, or Methods section.

n/a	Confirmed	
☐	☒	The exact sample size (*n*) for each experimental group/condition, given as a discrete number and unit of measurement
☐	☒	A statement on whether measurements were taken from distinct samples or whether the same sample was measured repeatedly
☐	☒	The statistical test(s) used AND whether they are one- or two-sided<br>*Only common tests should be described solely by name; describe more complex techniques in the Methods section.*
☐	☒	A description of all covariates tested
☐	☒	A description of any assumptions or corrections, such as tests of normality and adjustment for multiple comparisons
☐	☒	A full description of the statistical parameters including central tendency (e.g. means) or other basic estimates (e.g. regression coefficient) AND variation (e.g. standard deviation) or associated estimates of uncertainty (e.g. confidence intervals)
☐	☒	For null hypothesis testing, the test statistic (e.g. *F*, *t*, *r*) with confidence intervals, effect sizes, degrees of freedom and *P* value noted<br>*Give P values as exact values whenever suitable.*
☐	☒	For Bayesian analysis, information on the choice of priors and Markov chain Monte Carlo settings
☐	☒	For hierarchical and complex designs, identification of the appropriate level for tests and full reporting of outcomes
☐	☒	Estimates of effect sizes (e.g. Cohen's *d*, Pearson's *r*), indicating how they were calculated

Our web collection on statistics for biologists contains articles on many of the points above.

Software and code

Policy information about availability of computer code

Data collection	Analyses were conducted using R. The code reproducing our findings is available at https://doi.org/10.5281/zenodo.7327435 . All imaging data was available through The Cancer Imaging Archive. Imaging data from the Discovery Cohort can be found listed as Multi-center breast DCE-MRI data and segmentations from patients in the I-SPY 1/ACRIN 6657 trials (ISPY1) and imaging data from the Validation Cohort can be found listed as Multi-center breast DCE-MRI data and segmentations from patients in the I-SPY 1/ACRIN 6657 trials (ISPY1) and as Single site breast DCE-MRI data and segmentations from patients undergoing neoadjuvant chemotherapy (Breast-MRI-NACT-Pilot)<br>Gene expression information for the 100 women in the Discovery Cohort is available through the Gene Expression Ombinus under the accession number GSE2222628.
Data analysis	Analyses were conducted using R. The code reproducing our findings is available at https://doi.org/10.5281/zenodo.7327435

For manuscripts utilizing custom algorithms or software that are central to the research but not yet described in published literature, software must be made available to editors and reviewers. We strongly encourage code deposition in a community repository (e.g. GitHub). See the Nature Portfolio guidelines for submitting code & software for further information.

Data

Policy information about availability of data

All manuscripts must include a data availability statement. This statement should provide the following information, where applicable:

- Accession codes, unique identifiers, or web links for publicly available datasets
- A description of any restrictions on data availability
- For clinical datasets or third party data, please ensure that the statement adheres to our policy

The source data for all figures are available at https://doi.org/10.5281/zenodo.7327435. All imaging data was available through The Cancer Imaging Archive. Imaging data from the Discovery Cohort can be found listed as Multi-center breast DCE-MRI data and segmentations from patients in the I-SPY 1/ACRIN 6657 trials (ISPY1) and imaging data from the Validation Cohort can be found listed as Multi-center breast DCE-MRI data and segmentations from patients in the I-SPY 1/ACRIN 6657 trials (ISPY1) and as Single site breast DCE-MRI data and segmentations from patients undergoing neoadjuvant chemotherapy (Breast-MRI-NACT-Pilot)
Gene expression information for the 100 women in the Discovery Cohort is available through the Gene Expression Ombinus under the accession number GSE2222628.

Human research participants

Policy information about studies involving human research participants and Sex and Gender in Research.

Reporting on sex and gender	DCE-MR images of women enrolled in the ACRIN 6657/I-SPY1 trial, diagnosed with advanced invasive breast cancer from May 2002 through March 2006, were retrospectively analyzed. Per the inclusion criteria of ACRIN 6657/I-SPY 1, women diagnosed with stage 2 or 3 breast cancer were selected for the study and underwent anthracycline-cyclophosphamide NACT.
Population characteristics	Of the 222 trial participants with publicly available data, we retained the 143 women for whom both complete clinical data and T1 and T2 DCE-MR imaging were available. For analyses involving gene expression, we used the subset of 100 women for whom gene expression information was available through the Gene Expression Omnibus, under the accession number GSE2222628. Clinical and histopathologic data including age, hormone receptor (HR) status, human epidermal growth factor receptor 2 (HER2) status, and pCR status were available for each woman (Table 1). Functional tumor volume at T2 (FTV2), previously shown to have significant association with RFS21, was also calculated for each woman. RFS times were available, defined as time to recurrence (event), or time to death or last follow-up (censor). A validation cohort of 92 women was formed from the remaining 43 women from the original cohort (n=143) for whom gene expression data was not publicly available, and a separate dataset of 49 women from the publicly available Breast MRI NACT Pilot study This study had similar inclusion criteria as the I-SPY 1 trial, and participants underwent a similar treatment and imaging protocol as the I-SPY 1 trial. Clinical information on age, HR status, and HER2 status and 3-year RFS information was available for each woman in the validation cohort.
Recruitment	This study was a retrospective analysis using publicly available data.
Ethics oversight	All eligible patients selected for the I-SPY 1 TRIAL and Breast-MRI-NACT-Pilot study gave their written consent form. In the I-SPY 1 TRIAL, the Health Insurance Portability and Accountability Act–compliant protocol and the written consent were approved by the American College of Radiology Institutional Review Board and local-site institutional review boards.

Note that full information on the approval of the study protocol must also be provided in the manuscript.

Field-specific reporting

Please select the one below that is the best fit for your research. If you are not sure, read the appropriate sections before making your selection.

☒ Life sciences ☐ Behavioural & social sciences ☐ Ecological, evolutionary & environmental sciences

For a reference copy of the document with all sections, see nature.com/documents/nr-reporting-summary-flat.pdf

Life sciences study design

All studies must disclose on these points even when the disclosure is negative.

Sample size	No sample size calculation was performed. The analysis was limited to the samples available in the publicly available data.
Data exclusions	Of the 222 trial participants with publicly available data we retained the 143 women for whom both complete clinical data and T1 and T2 DCE-MR imaging were available. For analyses involving gene expression, we used the subset of 100 women for whom gene expression information was available through the Gene Expression Omnibus under the accession number GSE2222628
Replication	All models were evaluated using 5-fold cross validation and averaged over 100 replicates.
Randomization	For analyses involving gene expression, we used the subset of 100 women for whom gene expression information was available through the

Randomization	Gene Expression Omnibus under the accession number GSE2222628. The remaining women for whom gene expression information was not available were used in the validation cohort.
Blinding	All statistical analysis and cluster generation was done blinded to recurrence outcome.

Reporting for specific materials, systems and methods

We require information from authors about some types of materials, experimental systems and methods used in many studies. Here, indicate whether each material, system or method listed is relevant to your study. If you are not sure if a list item applies to your research, read the appropriate section before selecting a response.

Materials & experimental systems

n/a	Involved in the study
☒	☐ Antibodies
☒	☐ Eukaryotic cell lines
☒	☐ Palaeontology and archaeology
☒	☐ Animals and other organisms
☐	☒ Clinical data
☒	☐ Dual use research of concern

Methods

n/a	Involved in the study
☒	☐ ChIP-seq
☒	☐ Flow cytometry
☒	☐ MRI-based neuroimaging

Clinical data

Policy information about clinical studies

All manuscripts should comply with the ICMJE guidelines for publication of clinical research and a completed CONSORT checklist must be included with all submissions.

Clinical trial registration	ACRIN 6657
Study protocol	https://wiki.cancerimagingarchive.net/pages/viewpage.action?pageId=20643859
Data collection	ACRIN 6657 was designed as a prospective study to test MRI for ability to predict response to treatment and risk-of-recurrence in patients with stage 2 or 3 breast cancer receiving neoadjuvant chemotherapy (NACT). ACRIN 6657 was conducted as a companion study to CALGB 150007, a correlative science study evaluating tissue-based biomarkers in the setting of neoadjuvant treatment of breast cancer. Collectively, CALGB 150007 and ACRIN 6657 formed the basis of the multicenter Investigation of Serial Studies to Predict Your Therapeutic Response with Imaging and moLecular Analysis (I-SPY TRIAL) breast cancer trial, a study of imaging and tissue-based biomarkers for predicting pathologic complete response (pCR) and recurrence-free survival (RFS).Participant Eligibility and Enrollment: Criteria for inclusion were patients enrolling on CALGB 150007 with T3 tumors measuring at least 3 cm in diameter by clinical exam or imaging and receiving neoadjuvant chemotherapy with an anthracycline-cyclophosphamide regimen alone or followed by a taxane. Pregnant patients and those with ferromagnetic prostheses were excluded from the study. The study was open to enrollment from May 2002 to March 2006. 237 patients were enrolled, of which 230 met eligibility criteria.
Outcomes	The outcomes measured in this study were pathologic complete response (pCR) and recurrence-free survival (RFS). RFS times were available, defined as time to recurrence (event), or time to death or last follow-up (censor).
